# Effect of a Topical Thrombin–Carboxymethyl Starch Hemostatic Agent on Perioperative Hemoglobin Course: A Propensity Score-Matched Study

**DOI:** 10.3390/medicina62061142

**Published:** 2026-06-11

**Authors:** Dojoon Park, Hae-Seok Koh, Jeong Wook Moon, Youn-Ho Choi

**Affiliations:** Department of Orthopaedic Surgery, St. Vincent’s Hospital, College of Medicine, The Catholic University of Korea, 93, Joongbudae-ro, Paldal-gu, Suwon-si 16247, Gyeonggi-do, Republic of Korea; onedream1106@naver.com (D.P.); vincentos@naver.com (H.-S.K.); moon5648@gmail.com (J.W.M.)

**Keywords:** total knee arthroplasty, thrombin, carboxymethyl starch, topical hemostat, hemoglobin kinetics, postoperative anemia, perioperative blood management, propensity score matching

## Abstract

*Background and Objectives*: With contemporary blood management strategies substantially reducing transfusion rates after total knee arthroplasty (TKA), conventional endpoints such as transfusion incidence and estimated blood loss may have limited sensitivity for evaluating adjunctive hemostatic interventions. As postoperative anemia evolves dynamically over time, hemoglobin kinetics and cumulative anemia burden may offer more informative measures of treatment effect. This study evaluated whether implementation of a topical thrombin–carboxymethyl starch hemostatic agent within a standardized modern blood management protocol was associated with smaller early postoperative hemoglobin decline and lower cumulative anemia burden after TKA. *Materials and Methods*: In this single-center, retrospective, pre–post observational study, consecutive patients aged 50 years or older undergoing primary unilateral TKA for osteoarthritis before and after implementation of a thrombin–carboxymethyl starch topical hemostatic agent were compared. Perioperative management was otherwise standardized and unchanged. Patients were matched 1:1 using propensity scores derived from eight prespecified covariates. Co-primary endpoints were hemoglobin change from baseline to postoperative day 1 and day 2, and cumulative anemia burden quantified by the area under the hemoglobin-deficit curve from POD 0 to POD 13 was assessed as a key secondary endpoint. *Results*: Of 564 patients assessed for eligibility, 328 met the inclusion criteria, and 70 propensity score-matched pairs were included in the final analysis. Unless otherwise specified, the outcomes reported below were analyzed in these 70 matched pairs. In the matched cohort, the intervention group had a lesser hemoglobin decrease at postoperative day (POD) 1 than the control group (2.12 ± 0.97 vs. 2.42 ± 0.98 g/dL), corresponding to a paired mean difference of 0.30 g/dL (95% CI, 0.08–0.52; *p* = 0.008). The between-group difference at POD 2 was not statistically significant (paired mean difference, 0.15 g/dL; 95% CI, −0.03 to 0.33; *p* = 0.10). The area under the hemoglobin-deficit curve from POD 0 to POD 13 was lower in the intervention group (18.6 ± 5.2 vs. 21.3 ± 5.6 g/dL × day), with a paired mean difference of 2.7 g/dL × day (95% CI, 0.9–4.5; *p* = 0.004). Estimated total blood loss, formula-derived hidden blood loss, and transfusion rates did not differ significantly between groups. *Conclusions*: Use of a thrombin–carboxymethyl starch topical hemostatic agent was associated with modest attenuation of early postoperative hemoglobin decline and lower cumulative anemia burden after TKA, without significant differences in estimated blood loss or transfusion occurrence. Hemoglobin kinetics and cumulative anemia burden may provide complementary outcome measures in contemporary low-transfusion practice, although these findings should be interpreted cautiously given the observational design and low transfusion event rate.

## 1. Introduction

Perioperative blood management is a central component of total knee arthroplasty (TKA), with direct implications for postoperative recovery, anemia-related morbidity, and the overall patient experience [1]. Over the past decade, routine tranexamic acid (TXA) use, increasing adoption of drainless surgical protocols, and more restrictive transfusion thresholds have collectively reduced transfusion rates substantially in contemporary practice [2]. Consequently, the clinical context in which hemostatic strategies are evaluated has changed substantially [3]. In this modern low-transfusion environment, conventional endpoints such as transfusion incidence and estimated blood loss may no longer be sufficiently sensitive to detect modest differences between interventions [4]. This raises the question of whether transfusion-based metrics remain an adequate primary framework for assessing adjunctive hemostatic strategies in TKA [5,6]. When transfusion events are infrequent, the methodological challenge becomes how to meaningfully quantify the clinical value of such interventions [7]. A hemoglobin measurement at a single postoperative time point provides only a limited cross-sectional snapshot and may fail to capture the full magnitude and duration of postoperative hemoglobin decline [8]. Because perioperative anemia is better conceptualized as a dynamic exposure than a discrete event, measures that reflect hemoglobin kinetics over time may offer a more informative assessment [9]. One such measure is the area under the curve (AUC) of hemoglobin deficit, which integrates cumulative postoperative anemia burden across serial measurements [10,11]. At our institution, a thrombin–carboxymethyl starch topical hemostatic agent (ActiClot) was introduced into routine TKA practice within an otherwise standardized contemporary blood management protocol [12], providing a real-world opportunity to examine whether a topical hemostat confers measurable incremental benefit in an already optimized perioperative setting [13]. We therefore conducted a single-center, pre–post comparative observational study with propensity score matching to enhance comparability between patients managed before and after implementation of the topical thrombin–carboxymethyl starch hemostatic agent. The primary aim was to determine whether use of the topical thrombin–carboxymethyl starch hemostatic agent was associated with attenuated early postoperative hemoglobin decline and reduced cumulative anemia burden following TKA. To this end, we assessed hemoglobin change on postoperative day (POD) 1 and POD 2 and quantified cumulative anemia burden using the AUC of hemoglobin deficit. Although hemoglobin levels continue to decline beyond POD 1—with POD 2 reflecting a greater fall attributable to ongoing hidden blood loss and fluid redistribution—POD 1 represents the first actionable time point for perioperative decision-making, including transfusion evaluation and early mobilization assessment [14]. Accordingly, POD 1 and POD 2 were prespecified as co-primary endpoints, as each captures a distinct and complementary phase of early postoperative hemoglobin kinetics [14,15]. By evaluating hemoglobin kinetics and cumulative anemia burden alongside conventional outcomes, this study was designed to investigate whether incremental hemostatic benefit remains detectable in contemporary TKA practice, where transfusion-based endpoints have become progressively less discriminating.

## 2. Materials and Methods

### 2.1. Study Design and Setting

This single-center retrospective observational study was conducted at a university-affiliated tertiary referral hospital. Outcomes were compared between consecutive primary unilateral TKAs performed during a pre-implementation control period (May 2023 to August 2024) and a post-implementation intervention period (September 2024 to November 2025), following institutional adoption of a thrombin–carboxymethyl starch topical hemostatic agent (ActiClot; Theracion Biomedical, Seongnam-si, Gyeonggi-do, Republic of Korea).This study used a retrospective, nonrandomized pre–post observational design comparing patients who underwent primary unilateral TKA before and after institutional implementation of the topical thrombin–carboxymethyl starch hemostatic agent. Because treatment exposure was determined by calendar period rather than random allocation, the study was designed to evaluate associations between the topical hemostatic agent use and postoperative hemoglobin kinetics, rather than to establish definitive causal effects [16]. The topical hemostatic agent was introduced into routine clinical practice in September 2024. All procedures throughout both study periods were performed by a single surgeon using the same implant system; patellar resurfacing was not performed in any case. Apart from the implementation of the topical hemostatic agent, the operative technique, anesthesia pathway, and all aspects of perioperative management remained unchanged across study periods. These included tourniquet pressure (250 mmHg), preferred epidural anesthesia with conversion to general anesthesia when epidural anesthesia was not feasible, TXA administration, drain policy, thromboprophylaxis, analgesia protocol, transfusion thresholds, iron supplementation policy, and discharge and rehabilitation pathways. Data were obtained from an institutional arthroplasty registry derived from electronic medical records and routine postoperative clinic follow-up. The study was approved by the institutional review board; informed consent was waived given the retrospective nature of the study.

### 2.2. Participants

Consecutive patients aged 50 years or older who underwent primary unilateral TKA for osteoarthritis during the study period were screened for eligibility. Inclusion required a baseline hemoglobin measurement obtained on the day before surgery and postoperative hemoglobin values on both POD 1 and POD 2. Patients were excluded if they underwent simultaneous bilateral TKA, staged bilateral procedures, revision or conversion TKA, or any concomitant procedure at the time of index surgery. Additional exclusion criteria comprised known bleeding disorders, ongoing therapeutic anticoagulation, severe hepatic or renal disease, transfusion within four weeks before surgery, active infection, and a diagnosis other than primary osteoarthritis, including rheumatoid arthritis and post-traumatic osteoarthritis. For patients who underwent repeated procedures during the study period, only the index procedure was included in the analysis. For cohort-flow reporting, exclusion reasons were summarized using mutually exclusive categories. When more than one exclusion criterion was applicable, patients were assigned to the first applicable criterion in the predefined screening sequence.

### 2.3. Perioperative Blood Management Protocol

A standardized perioperative pathway was maintained throughout both study periods. Intravenous TXA was administered as a fixed 2 g dose prior to skin incision; topical and oral TXA were not used, and no TXA exceptions were made. Epidural anesthesia was the primary anesthetic approach, with conversion to general anesthesia when epidural anesthesia failed. A tourniquet was inflated before skin incision at 250 mmHg and released after final component implantation. Surgical drains were not used in any case. Pharmacologic thromboprophylaxis was not administered; intermittent pneumatic compression was used as mechanical prophylaxis in both periods. Postoperative analgesia followed the same institutional protocol, consisting primarily of celecoxib and acetaminophen with patient-controlled analgesia (PCA). Epidural PCA was used in patients managed under epidural anesthesia, whereas intravenous PCA and femoral nerve block were used when general anesthesia was required. Periarticular injection was not used.

Postoperative mobilization was initiated 24 h after surgery, and walker-assisted full weight-bearing ambulation was encouraged as tolerated. Continuous passive motion (CPM) and formal physiotherapy were initiated from POD 2, with CPM beginning at 0–60° and advanced as tolerated. The same discharge and rehabilitation pathway was used throughout both study periods. Restrictive transfusion criteria were applied consistently across both periods. Transfusion was considered for hemoglobin levels below 7 g/dL, or below 8 g/dL in patients with cardiovascular disease or symptomatic anemia, defined as dizziness, hypotension, tachycardia, or chest pain [17]. Transfusion decisions were made by the treating surgeon according to these predefined criteria. Intravenous iron was withheld until at least POD 7 and was thereafter administered only when clinically indicated at the discretion of the treating surgeon; oral iron was not prescribed. The postoperative laboratory schedule and laboratory equipment remained unchanged throughout the study period, and no major perioperative protocol changes were introduced other than the implementation of the topical hemostatic agent.

In the intervention period, the topical hemostatic agent was applied intra-articularly according to a standardized operative sequence documented in the operative records. After final component implantation, the tourniquet was released, followed by thorough irrigation and completion of suctioning. Immediately before capsular closure, a fixed volume of one kit (6 mL) was applied by the operating surgeon. The material was distributed over visible intra-articular bleeding surfaces, including the posterior capsule, medial and lateral gutters, suprapatellar pouch, and capsular bleeding surfaces. Capsular closure was then initiated after application of the material. All additional operative details relevant to perioperative blood loss remained unchanged throughout the study period, including all-cement fixation, layered wound closure, and no use of periarticular injection.

### 2.4. Laboratory Schedule and Data Collection

Baseline hemoglobin and hematocrit were measured in all patients on the day before surgery. Postoperative hemoglobin and hematocrit were obtained on POD 1, POD 2, POD 6, POD 8, and POD 13 according to a protocolized inpatient laboratory schedule, with an additional outpatient assessment at POD 28. Inpatient blood samples were collected in the early morning (approximately 06:00–07:00). Analyses were therefore performed using POD-defined time points; inpatient samples followed a fixed early-morning collection schedule. The POD28 outpatient assessment was scheduled within a routine follow-up window of POD28 ± 4 days (POD24–32).

Patient-reported fatigue was assessed using the PROMIS Fatigue T-score [18] administered by paper questionnaire. PROMIS data collection was incorporated into the routine clinical workflow from June 2024 onward; however, complete and systematically collected data were available only for patients during the intervention period. PROMIS Fatigue T-scores were collected at POD 6, POD 13, and POD 28 as part of routine postoperative follow-up during the intervention period. Because corresponding control-period data were incomplete and not systematically collected, PROMIS Fatigue was analyzed only within the intervention-period cohort as an exploratory descriptive outcome and was not used for between-group inference.

### 2.5. Outcomes

The study had two co-primary endpoints: hemoglobin change from baseline to POD 1 (POD1 ΔHb) and to POD 2 (POD2 ΔHb). ΔHb was defined as baseline hemoglobin minus postoperative hemoglobin at each postoperative time point; therefore, a larger positive ΔHb indicated a greater postoperative hemoglobin decrease. The area under the postoperative hemoglobin-deficit curve (AUC) was prespecified as a key secondary endpoint reflecting cumulative postoperative hemoglobin reduction over time [19]. Results were presented in a prespecified order for interpretive clarity, but this sequence was not intended as a formal hierarchical testing strategy; therefore, *p*-values were interpreted as nominal.

The key secondary endpoint was cumulative anemia burden quantified by the AUC of hemoglobin deficit from POD 0 to POD 13. An exploratory extension of this analysis evaluated AUC from POD 0 to POD 28. For AUC estimation, POD 0 was assigned a hemoglobin deficit of zero, corresponding to the preoperative baseline value. At each postoperative time point (POD 1, POD 2, POD 6, POD 8, POD 13, and POD 28), hemoglobin deficit was defined as max (0, baseline hemoglobin − postoperative hemoglobin). AUC was calculated using the trapezoidal method across POD-defined time points and is expressed in units of g/dL × day [20].

Additional secondary outcomes included estimated total blood loss (TBL), formula-derived hidden blood loss (HBL), and transfusion occurrence. Estimated TBL was calculated using a hematocrit-based method (Gross formula), incorporating estimated patient blood volume (Nadler formula) [21], preoperative hematocrit measured on the day before surgery, and postoperative hematocrit at POD 1 [22]. Because no postoperative drains were used, the recorded intraoperative blood loss was used as the only recorded external blood-loss component. Formula-derived HBL was calculated as estimated TBL minus recorded intraoperative blood loss. These blood-loss outcomes were interpreted as hematocrit-based estimates rather than directly observed blood-loss volumes.

Patient-reported fatigue was prespecified as an exploratory contextual outcome and was not included in between-group comparative analyses. PROMIS Fatigue T-scores were summarized descriptively at POD 6, POD 13, and POD 28 within the intervention cohort only. The association between PROMIS Fatigue at POD 13 and cumulative anemia burden (AUC of hemoglobin deficit from POD 0 to POD 13) was examined as an exploratory within-cohort analysis.

### 2.6. Propensity Score Matching

To reduce confounding inherent to the nonrandomized pre–post design, propensity score matching (PSM) was performed using eight covariates prespecified a priori on the basis of their consistent availability in the institutional registry and their clinical relevance to perioperative hemoglobin change, blood loss, and potential treatment allocation by period: age, sex, body mass index, ASA physical status class, diabetes mellitus, cardiovascular disease, baseline hemoglobin, and operative time. Propensity scores were estimated by logistic regression. Patients in the control and intervention groups were matched in a 1:1 ratio using nearest-neighbor matching without replacement, with a caliper width set at 0.2 standard deviations of the logit of the propensity score [23]. Post-matching covariate balance was assessed using standardized mean differences, with values below 0.10 considered indicative of acceptable balance [24].

### 2.7. Statistical Analysis

All primary and secondary between-group comparisons in the propensity score-matched cohort were conducted using matched-pair methods to account for the 1:1 matched design. Continuous outcomes were compared using paired *t*-tests as the primary analytic approach. Approximate normality of matched-pair differences was assessed using the Shapiro–Wilk test, and the results are summarized in Supplementary Table S1. If substantial departures from normality were identified, paired nonparametric sensitivity analyses were planned using the Wilcoxon signed-rank test. Binary outcomes with low event rates, including transfusion occurrence, were analyzed descriptively and compared using exact McNemar testing when applicable. Hemoglobin-based endpoints were presented in a prespecified interpretive order of ΔHb on POD 1, ΔHb on POD 2, and cumulative anemia burden quantified by POD 0–13 hemoglobin-deficit AUC. This ordering was used for interpretive clarity and was not intended as a formal hierarchical testing procedure. No formal multiplicity adjustment was applied; therefore, *p*-values were interpreted as nominal. Statistical significance was assessed using a two-sided alpha level of 0.05. Longitudinal postoperative hemoglobin changes were additionally evaluated using a linear mixed-effects model with fixed effects for treatment group, postoperative time point, and the group-by-time interaction, and a patient-level random intercept to account for within-patient correlation. Time was modeled as a categorical variable according to the predefined postoperative laboratory schedule. Model-based between-group differences were estimated as control minus intervention and reported with 95% confidence intervals.

Patient-reported fatigue was prespecified as an exploratory contextual outcome and was not included in between-group comparative analyses. PROMIS Fatigue T-scores were summarized descriptively over time within the intervention-period cohort. The association between POD 0–13 hemoglobin-deficit AUC and PROMIS Fatigue at POD 13 was examined using correlation analysis as an exploratory within-cohort analysis. No between-group comparisons or treatment-effect inferences were performed for PROMIS Fatigue.

Continuous variables were summarized as mean ± standard deviation to describe inter-patient variability. In the matched cohort, between-group treatment effects were summarized as paired mean differences with 95% confidence intervals and *p*-values. For longitudinal graphical display, hemoglobin trajectories were plotted as mean ± standard error to visualize the precision of the estimated group mean at each postoperative time point. Patient-level variability in hemoglobin values at each time point was provided separately as mean ± standard deviation in Supplementary Table S2.

### 2.8. Missing Data and Software

Availability of POD 1 and POD 2 hemoglobin values was required for inclusion in the analytic cohort; accordingly, analyses of the primary and co-primary endpoints were complete-case by design. For later postoperative time points and secondary outcomes, analyses were conducted using all available observations at each time point. Missingness at these later time points was minimal and showed no meaningful imbalance between groups.

All statistical computations were performed with SAS (version 9.4, SAS Institute, Inc., Cary, NC, USA, 2013) and the R program (version 3.2.4, R Core Team, Vienna, Austria, 2017), ensuring rigorous data handling and analysis integrity.

## 3. Results

### 3.1. Patient Characteristics and Covariate Balance

**Figure 1:**
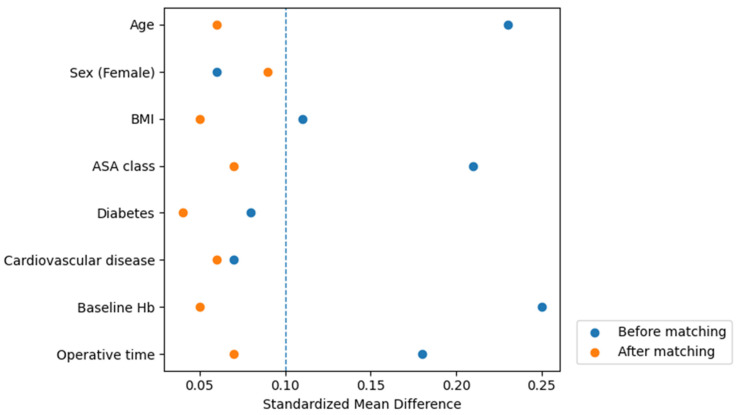
Love plot of standardized mean differences before and after propensity score matching. Blue dots represent standardized mean differences before matching, and orange dots represent those after matching. Propensity score matching was performed using nearest-neighbor matching without replacement and a caliper of 0.2 × SD of the logit of the propensity score. An SMD < 0.10 was considered indicative of adequate covariate balance.

During the study period, 287 patients in the control period and 277 patients in the intervention period were assessed for eligibility. After applying the predefined inclusion and exclusion criteria, 156 control-period patients and 172 intervention-period patients met the eligibility criteria. A total of 131 control-period patients and 105 intervention-period patients were excluded before matching, with detailed exclusion reasons shown in Figure S1. After 1:1 propensity score matching, 70 of 156 eligible control-period patients and 70 of 172 eligible intervention-period patients were retained in the final matched cohort, corresponding to matching retention rates of 44.9% and 40.7%, respectively. The remaining 86 control-period patients and 102 intervention-period patients were unmatched. Baseline characteristics before and after matching are presented in Table 1. After matching, covariate balance improved across all eight prespecified variables included in the propensity score model—age, sex, body mass index, ASA physical status class, diabetes mellitus, cardiovascular disease, baseline hemoglobin, and operative time—with all standardized mean differences below 0.10, indicating acceptable balance. Covariate balance before and after matching is shown in Figure 1.

### 3.2. Primary and Co-Primary Outcomes

Primary and co-primary outcomes in the matched cohort are summarized in Table 2. These analyses were performed in 70 matched pairs with available baseline, POD 1, and POD 2 hemoglobin values. The mean hemoglobin decrease at POD 1 was 2.42 ± 0.98 g/dL in the control group and 2.12 ± 0.97 g/dL in the intervention group, corresponding to a paired mean difference (control minus intervention) of 0.30 g/dL (95% CI, 0.08 to 0.52; *p* = 0.008). At POD 2, the mean hemoglobin decrease was 2.89 ± 1.05 g/dL in the control group and 2.74 ± 1.03 g/dL in the intervention group, with a paired mean difference of 0.15 g/dL (95% CI, −0.03 to 0.33; *p* = 0.10); the confidence interval crossed zero.

### 3.3. Hemoglobin Trajectories over Time

**Figure 2:**
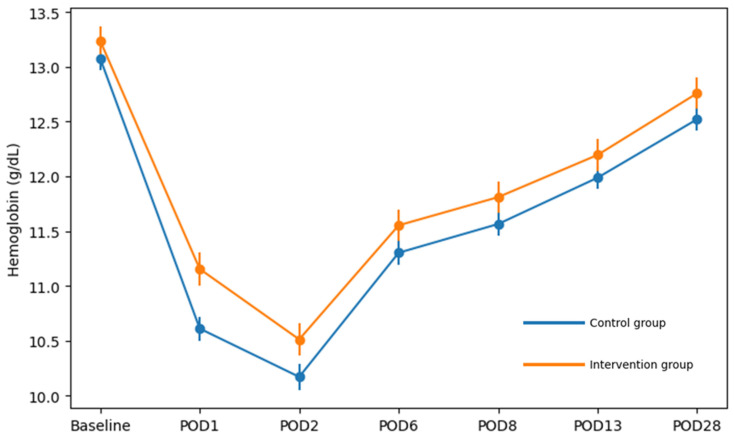
Hemoglobin trajectories over time (Baseline to POD 28) after propensity score matching. Blue lines represent the control group, and orange lines represent the intervention group. Values are presented as mean ± standard error. Laboratory tests were analyzed by postoperative day (POD); exact inpatient draw times were not collected. POD 28 assessments were obtained within the routine outpatient follow-up window of POD 28 ± 4 days. A repeated-measures mixed-effects model was additionally used to evaluate serial postoperative hemoglobin changes while accounting for within-patient correlation. Model-based estimates are provided in Supplementary Table S3.

Descriptive hemoglobin trajectories from baseline through POD 28 in the matched cohort are shown in Figure 2. In both groups, hemoglobin levels declined following surgery, reached a nadir around POD 2, and subsequently recovered over time. Between-group separation was greatest at POD 1 and diminished progressively at later postoperative time points, with both groups showing gradual recovery toward baseline values through POD 28.

A repeated-measures mixed-effects analysis was additionally performed to evaluate serial postoperative hemoglobin changes while accounting for within-patient correlation. The overall group-by-time interaction was not statistically significant (*p* = 0.18). However, model-based time-specific estimates were consistent with the primary matched-pair analysis. The estimated between-group difference in ΔHb was greatest at POD 1 (control − intervention, 0.31 g/dL; 95% CI, 0.06 to 0.56; *p* = 0.016) and attenuated at later time points, including POD 2 (0.16 g/dL; 95% CI, −0.06 to 0.38; *p* = 0.15). No statistically significant between-group difference was observed at POD 13 or POD 28. Detailed model-based estimates are provided in Supplementary Table S3. Among the 70 matched pairs included in POD 28–based analyses, POD 28 hemoglobin assessment was obtained at a median of POD 28 (IQR, POD 27–29; range, POD 24–32), within the routine outpatient follow-up window of POD 28 ± 4 days.

### 3.4. Key Secondary Outcomes

Key secondary outcomes are presented in Table 3. The AUC of hemoglobin deficit from POD 0 to POD 13 was 21.3 ± 5.6 g/dL × day in the control group and 18.6 ± 5.2 g/dL × day in the intervention group, corresponding to a paired mean difference of 2.7 g/dL × day (95% CI, 0.9 to 4.5; *p* = 0.004). For the exploratory AUC from POD 0 to POD 28, the mean value was 31.8 ± 8.4 g/dL × day in the control group and 29.5 ± 8.1 g/dL × day in the intervention group, with a paired mean difference of 2.3 g/dL × day (95% CI, −0.4 to 5.0; *p* = 0.09). Estimated total blood loss was 902 ± 312 mL in the control group and 879 ± 305 mL in the intervention group, with a paired mean difference of 23 mL (95% CI, −58 to 104; *p* = 0.58). Formula-derived hidden blood loss was 456 ± 221 mL in the control group and 438 ± 215 mL in the intervention group, with a paired mean difference of 18 mL (95% CI, −44 to 80; *p* = 0.57). Transfusion occurrence was assessed in all 70 matched pairs. Transfusion events were rare in both groups (1/70 [1.4%] in the control group versus 0/70 [0%] in the intervention group), and exact McNemar testing showed no evidence of a between-group difference (*p* = 1.00). The single transfusion event occurred in the matched control group on POD 3 for symptomatic anemia that met the predefined transfusion criteria. All key secondary outcomes were analyzed in the 70 matched pairs, unless otherwise specified. Formula inputs for estimated total blood loss and formula-derived hidden blood loss are summarized in Supplementary Table S4; no values were imputed.

### 3.5. Patient-Reported Fatigue

Exploratory findings for patient-reported fatigue within the intervention cohort are presented in Supplementary Figure S2. PROMIS Fatigue data were available only for the intervention-period cohort and were therefore analyzed descriptively without between-group comparison. PROMIS Fatigue T-scores declined from POD 6 to POD 28, consistent with the expected trajectory of postoperative recovery. Cumulative anemia burden from POD 0 to POD 13 was positively associated with PROMIS Fatigue T-score at POD 13 (r = 0.59, *p* < 0.001). As PROMIS Fatigue data were available only for the intervention period, these findings are descriptive and hypothesis-generating.

## 4. Discussion

In this propensity score-matched pre–post observational study conducted within a contemporary TKA blood management pathway—characterized by routine TXA use, drainless surgery, and restrictive transfusion practice—use of a topical thrombin–carboxymethyl starch hemostatic agent was associated with a modest but measurable smaller early postoperative hemoglobin decline [4,25]. Specifically, patients in the intervention period showed a smaller hemoglobin decrease at POD 1, the prespecified primary endpoint, along with a directionally consistent lower cumulative anemia burden over POD 0 to POD 13. In contrast, the between-group difference at POD 2 was less precisely estimated, and conventional blood loss metrics and transfusion occurrence did not differ materially between groups. Taken together, these findings suggest that in an already optimized low-transfusion setting, hemoglobin-based measures may provide potentially informative complementary outcomes for capturing modest perioperative differences that are not readily reflected by traditional hard endpoints [4,7,25].

Hemoglobin recovery after TKA is a dynamic process, and isolated postoperative measurements may not fully capture the overall extent and duration of perioperative anemia exposure [8,26,27,28]. Evaluating hemoglobin kinetics across multiple postoperative time points therefore provides a more integrated characterization of postoperative anemia than reliance on any single value [26]. Cumulative anemia burden, quantified as the area under the curve of hemoglobin deficit, incorporates both the magnitude and persistence of postoperative hemoglobin reduction and may thus complement conventional time-specific measures [8,29]. In the present study, the lower POD 0–13 cumulative anemia burden observed in the intervention group was directionally consistent with the POD 1 primary endpoint finding, despite attenuation of between-group differences at later individual time points. This pattern suggests that cumulative hemoglobin-based metrics may help detect modest perioperative differences that are not always apparent from isolated postoperative measurements alone [8].

The value of hemoglobin kinetics and hemoglobin-deficit AUC in this setting is primarily methodological and physiological. In contemporary TKA pathways using routine TXA, drainless surgery, and restrictive transfusion thresholds, transfusion events may be too infrequent to serve as a sensitive endpoint for adjunctive blood-conservation strategies. Hemoglobin-based endpoints may therefore help identify subtle perioperative physiological differences that would not be captured by transfusion occurrence alone. Nevertheless, the detection of such differences does not by itself establish that an adjunctive intervention improves recovery, reduces symptoms, or provides sufficient economic value to justify routine use.

Previous randomized trials and meta-analyses evaluating thrombin-based topical hemostatic agents in TKA have yielded mixed but interpretable findings, with observed effects varying according to both the endpoint assessed and the perioperative blood management context. Earlier meta-analyses reported generally favorable results, though not uniformly across outcomes: Wang et al. found significant reductions in hemoglobin decline and calculated total blood loss, but not in postoperative drainage volume or transfusion rate [30], whereas Fu et al. reported significant between-group differences in hemoglobin decline, total blood loss, drainage volume, and transfusion rate, all favoring Floseal [31]. More recently, an updated meta-analysis of eight studies involving 904 patients found that thrombin-based hemostatic matrix use was associated with reduced hemoglobin decline and a lower risk of allogeneic transfusion, while pooled differences in drainage volume and total blood loss were not statistically significant [13]. Individual studies within that analysis also demonstrated inconsistent results, with some randomized trials reporting no measurable benefit and others favoring hemostatic matrix use—a pattern attributed in part to differences in surgical technique, drainage protocols, and TXA use [32,33,34]. Our findings are best interpreted as broadly concordant with this body of evidence: hemoglobin-related outcomes showed modest between-group differences, whereas estimated blood-loss metrics and transfusion occurrence did not differ materially. However, direct comparison with prior studies should be made cautiously because background blood-management protocols were heterogeneous across reports, including differences in TXA use, drain practice, surgical technique, and transfusion thresholds. Such protocol-level differences may partly explain why thrombin-based topical hemostatic agents have shown variable findings across studies and may also influence which endpoints are most informative for capturing modest perioperative differences in contemporary low-transfusion settings.

The early POD1 association is biologically plausible because topical thrombin may enhance local fibrin clot formation, while the carboxymethyl starch matrix may support local absorption and clot stability at cancellous bone and periarticular soft-tissue bleeding surfaces. The attenuation by POD2 may reflect the increasing influence of fluid shifts, hemodilution, and equilibration of hidden blood loss on measured hemoglobin. Because local bleeding, wound hematoma, and fluid balance were not directly measured, this explanation remains inferential.

From a clinical perspective, the observed hemoglobin-based differences should be interpreted cautiously. Although use of the topical hemostatic agent was associated with a statistically significant reduction in POD1 hemoglobin decline and POD0–13 cumulative anemia burden, the absolute magnitudes of these differences were modest. A POD1 difference of approximately 0.30 g/dL is unlikely to be clinically decisive for most standard-risk patients whose postoperative hemoglobin levels remain well above conventional transfusion thresholds. In addition, no material differences were observed in estimated total blood loss, formula-derived hidden blood loss, or transfusion occurrence. Therefore, these findings should be interpreted primarily as measurable physiologic associations rather than definitive evidence of improved recovery, mobilization, fatigue, or other patient-centered benefits. Because this study did not include product cost, downstream resource utilization, or a formal cost-effectiveness analysis, whether this modest hematologic difference justifies the additional cost of routine use of the topical hemostatic agent cannot be determined from these data. The present findings do not support broad claims for routine use of topical thrombin-based hemostatic agents in all patients undergoing TKA within an already optimized blood-management pathway. Rather, the potential value of such agents may be greatest in selected patients with lower preoperative hemoglobin, borderline anemia, limited physiologic reserve, cardiovascular comorbidity, or higher anticipated perioperative blood loss, in whom even modest preservation of postoperative hemoglobin may have greater clinical relevance. This interpretation remains hypothesis-generating and requires prospective validation in predefined higher-risk subgroups. Further prospective studies incorporating mobilization, fatigue, functional recovery, length of stay, and cost-related outcomes are needed to determine whether modest improvements in hemoglobin kinetics have meaningful clinical or health-system consequences.

The exploratory PROMIS Fatigue analysis was included only to provide a patient-centered context for the hemoglobin-based findings [35]. Because PROMIS Fatigue data were systematically available only during the intervention period, these data cannot establish whether the topical hemostatic agent reduced postoperative fatigue compared with standard care. The observed within-cohort association between cumulative anemia burden and POD 13 fatigue should therefore be interpreted as hypothesis-generating evidence regarding the relationship between postoperative anemia burden and patient-reported fatigue, rather than as evidence of a treatment effect.

Several limitations of this study warrant acknowledgment. First, the nonrandomized pre–post design remains an important limitation. Although propensity score matching improved balance in measured baseline and operative covariates, treatment exposure was determined by calendar period rather than random allocation. Therefore, propensity score matching could not eliminate unmeasured temporal confounding, including secular changes, learning-curve effects, month-by-month case-mix variation, or subtle changes in perioperative practice. Accordingly, the observed differences should be interpreted as associations rather than definitive causal effects. Second, this was a single-center study conducted within a contemporary blood management pathway incorporating routine TXA use, drainless surgery, and restrictive transfusion thresholds; generalizability to settings with different perioperative practices or baseline transfusion risk may therefore be limited. Third, laboratory assessments were analyzed by postoperative day rather than exact sampling time, which may have introduced within-day measurement variability. In addition, postoperative hemoglobin values, estimated total blood loss, and formula-derived hidden blood loss may be influenced by perioperative fluid shifts and hemodilution; these outcomes should therefore be interpreted as laboratory-based or formula-derived estimates rather than direct measures of anatomical blood loss. Fourth, transfusion events were rare, limiting statistical power to detect differences in this conventional hard endpoint. Finally, patient-reported fatigue was exploratory and non-comparative, and is subject to multiple influencing factors beyond perioperative anemia. Within these limitations, the present findings should be interpreted as modest hemoglobin-based associations observed in a modern low-transfusion TKA setting, supporting the potential role of hemoglobin kinetics and cumulative anemia burden as complementary outcome measures rather than definitive evidence of a treatment effect.

## 5. Conclusions

In this propensity score-matched pre–post observational study, use of the topical thrombin–carboxymethyl starch hemostatic agent was associated with modestly smaller early postoperative hemoglobin decline and lower cumulative anemia burden after TKA performed within a drainless, low-transfusion blood-management pathway, while estimated blood-loss metrics and transfusion occurrence were not materially different between groups. These laboratory-based differences should not be interpreted as evidence of improved recovery, mobilization, fatigue, or other patient-centered benefits, nor do they establish routine clinical or economic value for all standard-risk patients. These findings support further evaluation of the topical hemostatic agent as a selective adjunct in patients with lower hematologic reserve or higher anticipated bleeding risk, while the observational design, small absolute effect size, and low event rate warrant cautious interpretation.

## Figures and Tables

**Table 1:** Baseline characteristics of the propensity score-matched cohort.

Variable	Control (*n* = 70)	Intervention (*n* = 70)	SMD
Age, years	69.9 ± 4.7	69.4 ± 4.6	0.06
Female sex, *n* (%)	62 (88.6%)	64 (91.4%)	0.09
Body mass index, kg/m^2^	26.6 ± 3.7	26.4 ± 3.6	0.05
ASA class, *n* (%)			
I	19 (27.1)	21 (30.0)	0.07
II	51 (72.9)	49 (70.0)	
III	0 (0.0)	0 (0.0)	
Diabetes mellitus, *n* (%)	16 (22.9%)	15 (21.4%)	0.04
Cardiovascular disease, *n* (%)	6 (8.6%)	5 (7.1%)	0.06
Baseline hemoglobin, g/dL	13.1 ± 1.0	13.2 ± 1.0	0.05
Operative time, min	90.8 ± 17.9	89.6 ± 18.3	0.07

Values are presented as mean ± standard deviation or number (%), as appropriate. ASA physical status class is presented as a number (%) by class and was entered into the propensity score model as an ordinal covariate. Standardized mean differences (SMDs) < 0.10 were considered indicative of adequate balance.

**Table 2:** Primary and co-primary outcomes (ΔHb at POD1 and POD2) in matched pairs.

Outcome	Control	Intervention	Paired Mean Difference (Control − Intervention)	95% CI	*p*-Value
ΔHb at POD1, g/dL	2.42 ± 0.98	2.12 ± 0.97	0.30	0.08 to 0.52	0.008
ΔHb at POD2, g/dL	2.89 ± 1.05	2.74 ± 1.03	0.15	−0.03 to 0.33	0.10

ΔHb was defined as baseline hemoglobin minus postoperative hemoglobin; therefore, a larger positive ΔHb indicates a greater postoperative hemoglobin decrease. Values are presented as mean ± standard deviation. Paired mean differences were calculated as control minus intervention; for ΔHb outcomes, a positive paired mean difference indicates a smaller postoperative hemoglobin decrease in the intervention group. Paired comparisons were performed using matched-pair methods. *p*-values are nominal.

**Table 3:** Secondary outcomes after propensity score matching.

Outcome	Control	Intervention	Paired Mean Difference (Control − Intervention)	95% CI	*p*-Value
AUC (Hb-deficit), POD0–13, g/dL × day	21.3 ± 5.6	18.6 ± 5.2	2.7	0.9 to 4.5	0.004
AUC (Hb-deficit), POD0–28, g/dL × day	31.8 ± 8.4	29.5 ± 8.1	2.3	−0.4 to 5.0	0.09
Estimated total blood loss (TBL), mL	902 ± 312	879 ± 305	23	−58 to 104	0.58
Formula-derived hidden blood loss (HBL), mL	456 ± 221	438 ± 215	18	−44 to 80	0.57
Any transfusion, *n* (%)	1 (1.4%)	0 (0%)	–	–	1.00 †
RBC units transfused	0.01 ± 0.12	0.00 ± 0.00	–	–	–

Values are presented as mean ± standard deviation unless otherwise indicated. Unless otherwise specified, analyses were performed in the 70 matched pairs. Paired mean differences were calculated as control minus intervention. AUC was calculated using the trapezoidal method and expressed as g/dL × day. Estimated total blood loss and formula-derived hidden blood loss were calculated using hematocrit-based formulas and should be interpreted as estimates rather than directly observed blood-loss volumes. Continuous outcomes were compared using paired *t*-tests in the matched cohort, and transfusion occurrence was compared using exact McNemar testing. † Exact McNemar test. *p*-values are nominal.

## Data Availability

Data sharing is not applicable.

## Supplementary Materials

The following supporting information can be downloaded at https://www.mdpi.com/article/10.3390/medicina62061142/s1: Figure S1: Flow diagram of patient selection and propensity score matching; Figure S2: Patient-reported postoperative fatigue during the intervention period; Table S1: Normality assessment of matched-pair differences; Table S2: Hemoglobin values by group and postoperative time point; Table S3: Mixed-effects model estimates for serial postoperative hemoglobin change; Table S4: Formula inputs for estimated blood-loss calculations.

## References

[1] Zhang F.Q., Yang Y.Z., Li P.F., Ma G.R., Zhang A.R., Zhang H., Guo H.Z. (2024). Impact of preoperative anemia on patients undergoing total joint replacement of lower extremity: A systematic review and meta-analysis. J. Orthop. Surg. Res..

[2] Sawant S., Deshpande S.V., Wamborikar H., Jadawala V.H., Suneja A., Goel S., Patel V. (2024). The Impact of Tranexamic Acid on Blood Loss Management in Primary Total Knee Arthroplasty: A Comprehensive Review. Cureus.

[3] Kholmukhamedov A., Subbotin D., Gorin A., Ilyassov R. (2025). Anticoagulation Management: Current Landscape and Future Trends. J. Clin. Med..

[4] Fillingham Y.A., Ramkumar D.B., Jevsevar D.S., Yates A.J., Bini S.A., Clarke H.D., Schemitsch E., Johnson R.L., Memtsoudis S.G., Sayeed S.A. (2019). Tranexamic acid in total joint arthroplasty: The endorsed clinical practice guides of the American Association of Hip and Knee Surgeons, American Society of Regional Anesthesia and Pain Medicine, American Academy of Orthopaedic Surgeons, Hip Society, and Knee Society. Reg. Anesth. Pain Med..

[5] Maman D., Nandakumar M., Hirschmann M.T., Ofir H., Haddad M., Samir B., Steinfeld Y., Berkovich Y. (2025). Blood transfusion in total knee arthroplasty and total hip arthroplasty: A nationwide study of complications, costs and predictive modelling. J. Exp. Orthop..

[6] Chen J., Zhong X., Zhai Y., Zhao C., Lan J., Chen L., Xia Z. (2025). Clinical prediction models for postoperative blood transfusion after total knee arthroplasty: A systematic review and meta-analysis. BMC Musculoskelet. Disord..

[7] Choi K.Y., Koh I.J., Kim M.S., Kim C., In Y. (2022). Intravenous Ferric Carboxymaltose Improves Response to Postoperative Anemia Following Total Knee Arthroplasty: A Prospective Randomized Controlled Trial in Asian Cohort. J. Clin. Med..

[8] Crispell E.H., Trinh J., Warner M.A. (2023). Postoperative anaemia: Hiding in plain sight. Best. Pract. Res. Clin. Anaesthesiol..

[9] Shander A., Corwin H.L., Meier J., Auerbach M., Bisbe E., Blitz J., Erhard J., Faraoni D., Farmer S.L., Frank S.M. (2023). Recommendations From the International Consensus Conference on Anemia Management in Surgical Patients (ICCAMS). Ann. Surg..

[10] Alzu’bi M., Bawa’neh H., Alshorman A., Alrawabdeh J., Odeh N., Hamadneh Y., AlAdwan M., Odeh M., Awidi A. (2023). Defining an optimal cut-off point for reticulocyte hemoglobin as a marker for iron deficiency anemia: An ROC analysis. PLoS ONE.

[11] Kolin D.A., Lyman S., Della Valle A.G., Ast M.P., Landy D.C., Chalmers B.P. (2023). Predicting Postoperative Anemia and Blood Transfusion Following Total Knee Arthroplasty. J. Arthroplast..

[12] Kim H.J., Lee S.K., Ko Y.J., Jeon S.H., Kim E.J., Kwon O.H., Cho Y.H. (2024). Novel Flowable Hemostatic Agent ActiClot: Efficacy and Safety Assessment in Rat and Porcine Models. J. Clin. Med..

[13] Park J.W., Kim T.W., Chang C.B., Han M., Go J.J., Park B.K., Jo W.L., Lee Y.K. (2023). Effects of Thrombin-Based Hemostatic Agent in Total Knee Arthroplasty: Meta-Analysis. J. Clin. Med..

[14] Khalfaoui M.Y., Godavitarne C., Wilkinson M.C. (2017). Optimal Timing for Hemoglobin Concentration Determination after Total Knee Arthroplasty: Day 1 versus Day 2. Knee Surg. Relat. Res..

[15] Nakamori E., Shigematsu K., Higashi M., Yamaura K. (2021). Postoperative Noninvasive Hemoglobin Monitoring Is Useful to Prevent Unnoticed Postoperative Anemia and Inappropriate Blood Transfusion in Patients Undergoing Total Hip or Knee Arthroplasty: A Randomized Controlled Trial. Geriatr. Orthop. Surg. Rehabil..

[16] Nianogo R.A., Benmarhnia T., O’Neill S. (2023). A comparison of quasi-experimental methods with data before and after an intervention: An introduction for epidemiologists and a simulation study. Int. J. Epidemiol..

[17] Carson J.L., Stanworth S.J., Guyatt G., Valentine S., Dennis J., Bakhtary S., Cohn C.S., Dubon A., Grossman B.J., Gupta G.K. (2023). Red Blood Cell Transfusion: 2023 AABB International Guidelines. JAMA.

[18] Yang M., Keller S., Lin J.S. (2019). Psychometric properties of the PROMIS^®^ Fatigue Short Form 7a among adults with myalgic encephalomyelitis/chronic fatigue syndrome. Qual. Life Res..

[19] Dmitrienko A., Tamhane A.C. (2007). Gatekeeping procedures with clinical trial applications. Pharm. Stat..

[20] Chiou W.L. (1978). Critical evaluation of the potential error in pharmacokinetic studies of using the linear trapezoidal rule method for the calculation of the area under the plasma level–time curve. J. Pharmacokinet. Biopharm..

[21] Nadler S.B., Hidalgo J.H., Bloch T. (1962). Prediction of blood volume in normal human adults. Surgery.

[22] Lin Y.M., Yu C., Xian G.Z. (2024). Calculation methods for intraoperative blood loss: A literature review. BMC Surg..

[23] Kane L.T., Fang T., Galetta M.S., Goyal D.K.C., Nicholson K.J., Kepler C.K., Vaccaro A.R., Schroeder G.D. (2020). Propensity Score Matching: A Statistical Method. Clin. Spine Surg..

[24] Nguyen T.L., Collins G.S., Spence J., Daurès J.P., Devereaux P.J., Landais P., Le Manach Y. (2017). Double-adjustment in propensity score matching analysis: Choosing a threshold for considering residual imbalance. BMC Med. Res. Methodol..

[25] Carson J.L., Guyatt G., Heddle N.M., Grossman B.J., Cohn C.S., Fung M.K., Gernsheimer T., Holcomb J.B., Kaplan L.J., Katz L.M. (2016). Clinical Practice Guidelines From the AABB: Red Blood Cell Transfusion Thresholds and Storage. JAMA.

[26] Gómez-Ramirez S., Jericó C., Muñoz M. (2019). Perioperative anemia: Prevalence, consequences and pathophysiology. Transfus. Apher. Sci..

[27] Ke C., Tian N., Zhang X., Chen M. (2020). Changes in perioperative hemoglobin and hematocrit in patients undergoing total knee arthroplasty: A prospective observational study of optimal timing of measurement. J. Int. Med. Res..

[28] Cho M.R., Jun C.M., Song S.K., Choi W.K. (2021). Natural course of hemoglobin level after total knee arthroplasty and the benefit of tranexamic acid injection in the joint. Medicine.

[29] Duque-Sosa P., Martínez-Urbistondo D., Echarri G., Callejas R., Iribarren M.J., Rábago G., Monedero P. (2017). Perioperative hemoglobin area under the curve is an independent predictor of renal failure after cardiac surgery. Results from a Spanish multicenter retrospective cohort study. PLoS ONE.

[30] Wang C., Han Z., Zhang T., Ma J.X., Jiang X., Wang Y., Ma X.L. (2014). The efficacy of a thrombin-based hemostatic agent in primary total knee arthroplasty: A meta-analysis. J. Orthop. Surg. Res..

[31] Fu X., Tian P., Xu G.J., Sun X.L., Ma X.L. (2017). Thrombin-Based Hemostatic Agent in Primary Total Knee Arthroplasty. J. Knee Surg..

[32] Kim H.J., Fraser M.R., Kahn B., Lyman S., Figgie M.P. (2012). The efficacy of a thrombin-based hemostatic agent in unilateral total knee arthroplasty: A randomized controlled trial. J. Bone Jt. Surg. Am..

[33] Velyvis J.H. (2015). Gelatin matrix use reduces postoperative bleeding after total knee arthroplasty. Orthopedics.

[34] Yen S.H., Lin P.C., Wu C.T., Wang J.W. (2021). Comparison of Effects of a Thrombin-Based Hemostatic Agent and Topical Tranexamic Acid on Blood Loss in Patients with Preexisting Thromboembolic Risk Undergoing a Minimally Invasive Total Knee Arthroplasty. A Prospective Randomized Controlled Trial. Biomed. Res. Int..

[35] Zargar-Shoshtari K., Hill A.G. (2009). Postoperative fatigue: A review. World J. Surg..

