# Supplementary material for: Effect of a Topical Thrombin–Carboxymethyl Starch Hemostatic Agent on Perioperative Hemoglobin Course: A Propensity Score-Matched Study
Source: Medicina (Kaunas). 2026 Jun 11;62(6):1142. doi: 10.3390/medicina62061142 (PMC13303010; doi:10.3390/medicina62061142)
Supplement: Supplementary file 1 [file medicina-62-01142-s001.zip › medicina-4244063-supplementary.pdf]

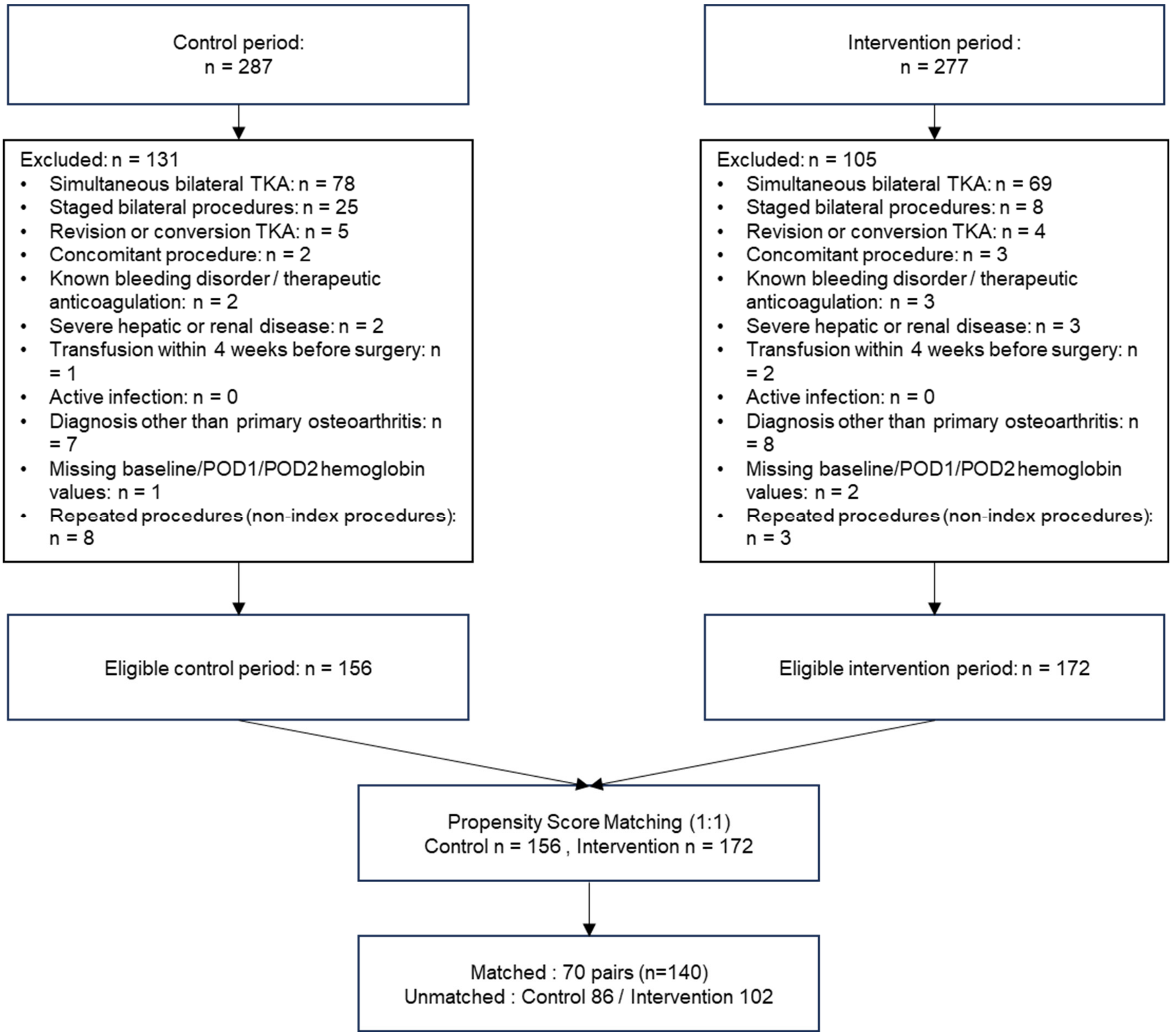

Supplementary Figure S1. Flow diagram of patient selection and propensity score matching.

Exclusion reasons were summarized using mutually exclusive categories for cohort-flow reporting. A total of 131 control-period patients and 105 intervention-period patients were excluded before matching. Among eligible patients, matching retained 70 of 156 control-period patients (44.9%) and 70 of 172 intervention-period patients (40.7%); 86 and 102 eligible patients, respectively, remained unmatched.

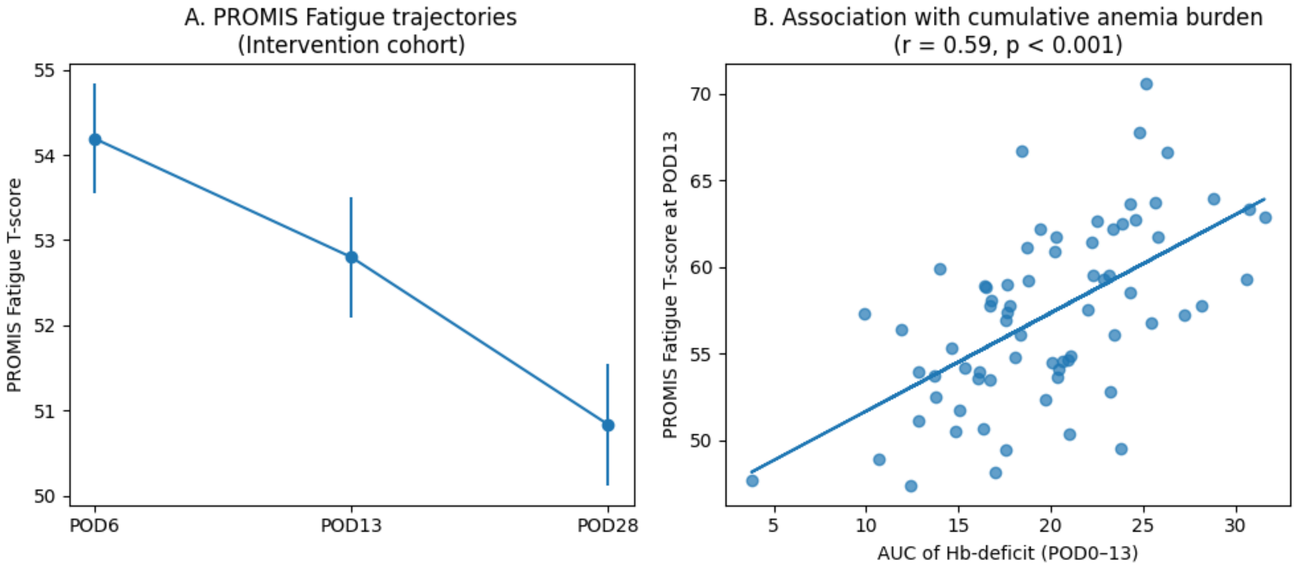

Supplementary Figure S2. Patient-reported postoperative fatigue during the intervention period.
(A) Trajectories of PROMIS Fatigue T-scores over time (postoperative days 6, 13, and 28) within the intervention cohort, presented as mean ± standard error. (B) Association between cumulative anemia burden, quantified by the area under the curve (AUC) of hemoglobin deficit from POD0 to POD13, and PROMIS Fatigue T-scores at POD13 within the intervention cohort. PROMIS Fatigue was collected during the intervention period only and analyzed as an exploratory outcome.

Supplementary Table S1. Normality assessment of matched-pair differences.

.Endpoint	Available matched pairs	Shapiro–Wilk W	p-value	Primary test	Sensitivity test
ΔHb POD 1	70	0.981	0.39	Paired t-test	Not required
ΔHb POD 2	70	0.976	0.22	Paired t-test	Not required
POD 0–13 Hb-deficit AUC	70	0.984	0.51	Paired t-test	Not required
POD 0–28 Hb-deficit AUC	70	0.972	0.14	Paired t-test	Not required
Estimated total blood loss	70	0.969	0.09	Paired t-test	Not required
Formula-derived hidden blood loss	70	0.971	0.12	Paired t-test	Not required

Shapiro–Wilk tests were performed on matched-pair differences calculated as control minus intervention. No substantial departures from normality were identified for the main continuous endpoints; therefore, paired t-tests were retained as the primary analytic approach and no additional nonparametric sensitivity test was required. AUC = area under the curve; Hb = hemoglobin; POD = postoperative day.

Supplementary Table S2. Hemoglobin values by group and postoperative time point.

Time point	Control Hb, mean ± SD, g/dL	Intervention Hb, mean ± SD, g/dL	Available matched pairs
Baseline	13.10 ± 1.00	13.20 ± 1.00	70
POD 1	10.68 ± 1.10	11.08 ± 1.10	70
POD 2	10.21 ± 1.10	10.46 ± 1.10	70
POD 6	11.50 ± 1.00	11.87 ± 1.00	70
POD 8	11.85 ± 0.95	12.23 ± 0.95	70
POD 13	12.11 ± 0.90	12.30 ± 0.90	70
POD 28	12.69 ± 0.85	12.65 ± 0.85	70

Values are presented as mean ± standard deviation. Hb = hemoglobin; POD = postoperative day. Available matched pairs indicate pairs with hemoglobin values available at each time point. POD 28 hemoglobin assessments were obtained within the routine outpatient follow-up window of POD 28 ± 4 days (POD 24–32); the actual assessment timing was median POD 28 (IQR, POD 27–29; range, POD 24–32).

Supplementary Table S3. Mixed-effects model estimates for serial postoperative hemoglobin change.

Time point	Estimated ΔHb difference, Control − Intervention	95% CI	p-value
POD 1	0.31 g/dL	0.06 to 0.56	0.016
POD 2	0.16 g/dL	−0.06 to 0.38	0.15
POD 6	0.23 g/dL	−0.03 to 0.49	0.08
POD 8	0.21 g/dL	−0.05 to 0.47	0.11
POD 13	0.08 g/dL	−0.16 to 0.32	0.51
POD 28	−0.12 g/dL	−0.36 to 0.12	0.32

Values represent model-based estimated between-group differences in hemoglobin change from baseline, calculated as control minus intervention. Positive values indicate a smaller postoperative hemoglobin decrease in the intervention group. Estimates were derived from a linear mixed-effects model including fixed effects for treatment group, postoperative time point, and the group-by-time interaction, with a patient-level random intercept to account for within-patient correlation. Time was modeled as a categorical variable. P-values are nominal.

Supplementary Table S4. Formula inputs for estimated blood-loss calculations.

Variable	Control group	Intervention group	Available matched pairs
Preoperative hematocrit, %	39.2 ± 3.0	39.5 ± 3.1	70
POD 1 hematocrit, %	31.8 ± 3.2	32.2 ± 3.1	70
Estimated patient blood volume, mL	4300 ± 610	4310 ± 600	70
Recorded intraoperative blood loss, mL	446 ± 180	441 ± 175	70
Estimated total blood loss, mL	902 ± 312	879 ± 305	70
Formula-derived hidden blood loss, mL	456 ± 221	438 ± 215	70

Values are presented as mean ± standard deviation. POD = postoperative day. Estimated patient blood volume was calculated using the Nadler formula. Estimated total blood loss was calculated using the Gross formula based on preoperative and POD 1 hematocrit values. Because no postoperative drains were used, recorded intraoperative blood loss was used as the only recorded external blood-loss component. Formula-derived hidden blood loss was calculated as estimated total blood loss minus recorded intraoperative blood loss. No values were imputed.
